# Supplementary material for: Virulence Potential of a Multidrug-Resistant Escherichia coli Strain Belonging to the Emerging Clonal Group ST101-B1 Isolated from Bloodstream Infection
Source: Microorganisms. 2020 May 30;8(6):827. doi: 10.3390/microorganisms8060827 (PMC7355805; doi:10.3390/microorganisms8060827)
Supplement: Supplementary file 1 [file microorganisms-08-00827-s001.zip › Suppl/Figure S5.pdf]

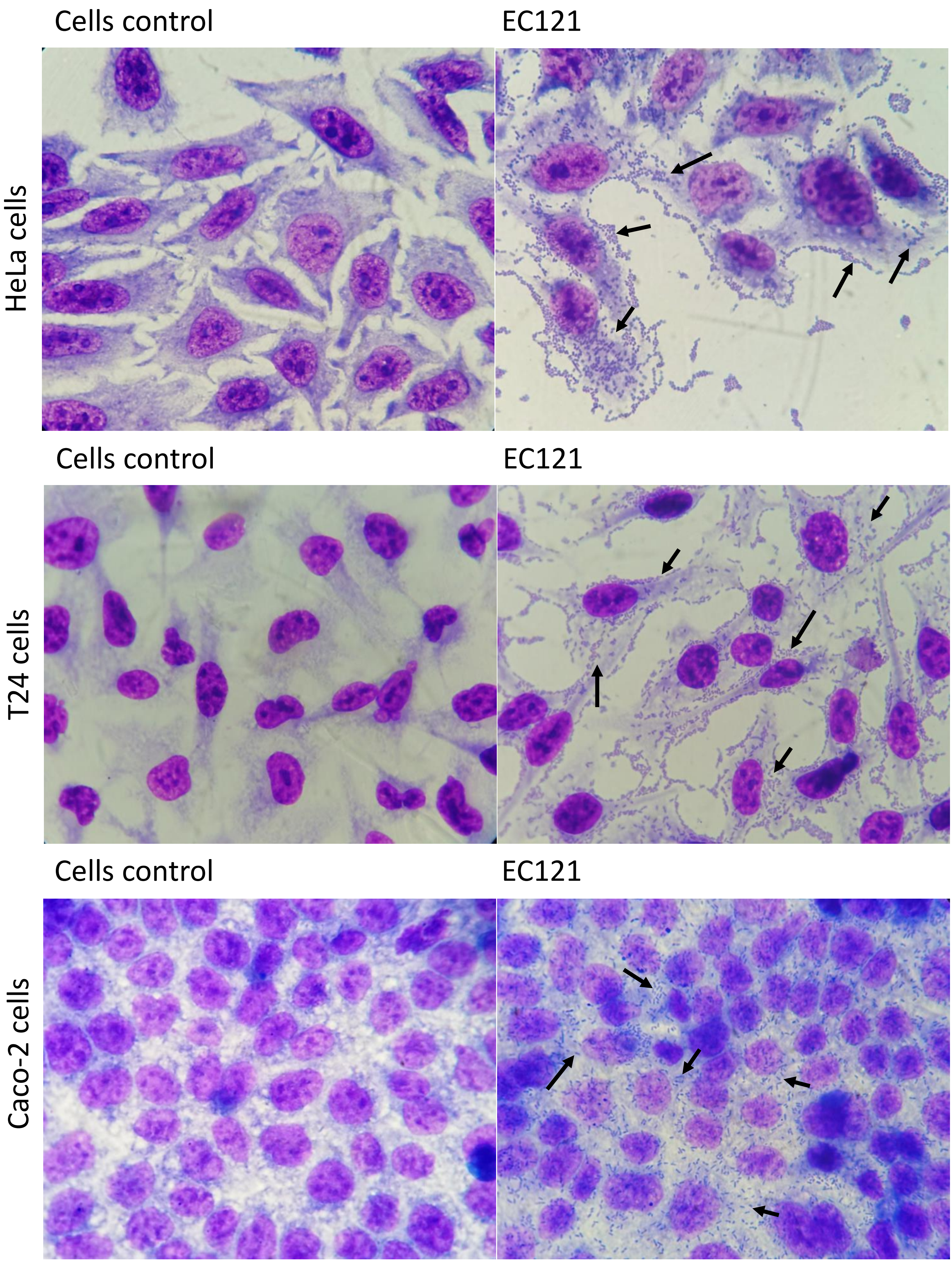

Figure S5 – Qualitative interaction assay of EC121 with HeLa, T24, and Caco-2 epithelial cells. The assays were performed in 3h of incubation at 37 °C, in the absence of D-mannose. The black arrows are pointing to some bacterial-epithelial cell interaction sites.
