# Supplementary material for: Virulence Potential of a Multidrug-Resistant Escherichia coli Strain Belonging to the Emerging Clonal Group ST101-B1 Isolated from Bloodstream Infection
Source: Microorganisms. 2020 May 30;8(6):827. doi: 10.3390/microorganisms8060827 (PMC7355805; doi:10.3390/microorganisms8060827)
Supplement: Supplementary file 1 [file microorganisms-08-00827-s001.zip › Suppl/Table S2 T5SS.docx]

| Table S2 – Autotransporters proteins identified in EC121 via MacSyFinder | | | |
| --- | --- | --- | --- |
| Virulence factor related ^a^ | Gene | Involved in | Reference |
| TA^*^ Adhesin EhaG | *ehaG* | Mediates biofilm formation, and adherence to extracellular matrix components, promotes adhesion to intestinal cells | Totsika et al 2012 |
| AIDA-I family autotransporter YfaL | *yfaL* | Not known – outer membrane protein | Marani et al 2006 |
| Autotransporter adhesin | *misL* | Involved in adherence, biofilm formation and invasion in *Salmonella enterica* serovar Typhimurium | Wang et al 2018 |
| Putative autotransporter protein |  | Not known – not described |  |
| Uncharacterized protein YcgV | *ycgV* | Involved in biofilm formation | Roux et al 2005 |
| Uncharacterized protein YaiT | *yaiT* | Not known – outer membrane protein |  |
| Uncharacterized lipoprotein YdeK | *ydeK* | Not known – in K-12 the sequence is disrupted. In EC121 the sequence is 488 amino acids length longer |  |
| Uncharacterized protein YhjY | *yhjY* | Involved in bacterial adhesion and biofilm formation | Vlasbom et al 2015 |
| Uncharacterized protein YuaQ | *yuaQ* | Not known – outer membrane protein |  |

^a^ The Uncharacterized outer membrane protein YejO, identified as an autotransporter protein was identified disrupted in EC121.

* Trimeric Autotransporter protein
