# Supplementary material for: Virulence Potential of a Multidrug-Resistant Escherichia coli Strain Belonging to the Emerging Clonal Group ST101-B1 Isolated from Bloodstream Infection
Source: Microorganisms. 2020 May 30;8(6):827. doi: 10.3390/microorganisms8060827 (PMC7355805; doi:10.3390/microorganisms8060827)
Supplement: Supplementary file 1 [file microorganisms-08-00827-s001.zip › Suppl/Table S3.docx]

**Table S3 - Completed Genetic cluster identified in EC121 strain and their predicted association with virulence.**

| Trait | Virulence factor related | Genetic cluster | Association with virulence | Reference |
| --- | --- | --- | --- | --- |
| Adhesin | intimin-like adhesin FdeC | *eaeH* | Colonization of urinary tract infection and biofilm formation | [^1^] |
|  | Pix fimbriae | *pixBAHCDJFG* | Pathogenicity of some UPEC strains | [^2^] |
|  | Ygi fimbriae | *ygiLyqiGHI* | Adhesion to urinary tract and biofilm formation and better fitness response in UPEC | [^3^] |
|  | Long polar fimbriae 1 (LPF) | *lpfABCDE* | Intestinal adhesion and colonization | [^4^] |
|  | Yad fimbriae | *yadCKLM*-*htrE*-*yadVN* | Bacteria-bacteria adhesion and biofilm formation, adherence to bladder | [^3^] |
|  | Sfm fimbriae | *sfmACDHF* | Adhesion to bladder cells and biofilm formation | [^5^] |
|  | Yeh fimbriae | *yehABCDE* | adhesion to various surfaces in specific environmental niches | [^5^] |
|  | Ybg fimbriae | *ybgOPQD* | Unknown | [^5^] |
|  | Yra fimbriae | *yraHIJK* | Adhesion to bladder cells and biofilm formation | [^5^] |
|  | Type 1 fimbriae | *fimBEAICDFGH* | Adhesion and invasion to epithelial cell and biofilm formation | [^6^] |
|  | Yde fimbriae | *fimACDydeSfimGH* | Putative adhesin – Unknown |  |
|  | *E. coli* comum pilus | *ecpRABCDE* | Adherence to epithelial cells | [^7^] |
|  | Hemorragic coli pili | *hcpABC* | Mediate adhesion, invasion, biofilm formation and twitching motility | [^8^] |
|  | Yfc fimbriae | *yfcOPQRSUV* | Adherence to many surfaces, mediates adhesion to bladder cells and biofilm formation | [^5^] |
|  | TA^*^ Adhesin EhaG | *ehaG* | Mediates biofilm formation, and adherence to extracellular matrix components, promotes adhesion to intestinal cells | [^9^] |
| Adhesin | Autotransporter adhesin | *Z0309* | Described in O157:H7 it’s required for intestinal colonization in calves | [^10^] |
|  | *E. coli* lamimin-binding fimbriae | *elfADCG-ycbUVF* | Involved in adherence to epithelial cells and extracellular matrix | [^11^] |
|  | Curli fimbriae | *csgCAB - csgDEFG* | Mediates biofilm formation at 37 °C and fibronectin binding | [^12^] |
| Bacteriocin | Colicin M | *cma* | Colicin, active against other bacteria, promotes colonization by eliminating niche competition | [^13^] |
| Immune evasion | Superoxide dismutase | *sodB* | Confers resistance to phagocytose | [^14^] |
|  | Thioredoxin 1 | *trxA* | Response against oxidative stresses | [^15^] |
|  | Incresed serum survival | *iss* | Promotes serum resistance | [^16^] |
|  | Mig-14 ortholog and Hemolisin F | *mig-14hlyF* | Involved in production of outer membrane vesicle, cleaving of CAMPs and escape of macrophage phagosome | [^17,18^] |
| Intracellular Spread | Outer membrane phospholipid binding lipoprotein MlaA | *vacJ* | Intracellular spread in *Shigella* and EIEC, responsible for outer membrane proteins and lipids correct organization | [^19^] |
| Invasion | intimin-like protein YchO | *ychO* | Adhesion, invasion and biofilm formation in APEC | [^20^] |
|  | IbeB (CusC) | *cusSRCFBA* | The operon acts as cation efflux pump to Cu and Ag, the protein CusC (IbeB) also acts in the invasion of blood brain barrier promoting meningitis | [^21,22^] |
|  | Outer membrane protein A | *ompA* | Involved in bacterial invasion of the brain microvascular endothelial cells, that leads to meningitis | [^22,23^] |
| Invasion | Phosphoethanolamine transferase EptC | *yijP* (*eptC*) | Involved in blood brain barrier invasion | [^24^] |
|  | FkpA precursor | *fkpA* | Involved in intracellular survival | [^25^] |
| Iron transport system | Salmonella iron transport system | *sitABCD* | Iron acquisition system | [^26^] |
|  | Salmochelin | *iroBCDEN* | Iron acquisition sytem | [^27^] |
|  | Ferric hydroxamate | *fhuACDB* | hydroxamate-type siderophore | [^28^] |
| Protease/immune evasion | Outer membrane protein T - plasmidial variant | *ompT_p_^a^* | Protease, acts in immune evasion by cleaving cationic antimicrobial peptides (CAMPs) | [^18^] |
|  | Outer membrane protein T - chromosomal variant | *ompT_c_^a^* | Protease that can cleave T7 RNA polymerase, ferric enterobactin receptor protein (FEP), antimicrobial peptide protamine and other proteins. | [^29^] |
| Regulator/ virulence expression | Two-component system QseBC | *qseBC* | Host sensing and regulation of virulence factors expression | [^30,31^] |
| Regulator/immune system evasion | Two-component system PhoPQ | *phoPQ* | Host sensing and regulation of expression of virulence genes related ExPEC evasion of immune system | [^18^] |
| Regulator/resistance and bacterial survival | Two-component system EvgAS | *evgAS* | Two component system involved in regulation of efflux pumps and acid resistance | [^32^] |
| HTH Regulator/immune evasion | SlyA | *slyA* | Involved in regulation of virulence genes and genes involved in scape from macrophages and resistance to oxidative stress | [^33–35^] |
| Regulator | Salmonella invasion regulator SirA | *sirA/uvrY* | Regulator involved in intracellular survival in *Salmonella* | [^36^] |
| Regulator/Kinase/ Virulence expression | GTP pyrophosphokinase | *relA* | Involved in the biofilm formation, regulation and bacterial persistence | [^37,38^] |
| Regulation/virulence | Two-component System DsbAB | *dsbAB* | Acts promoting disulfides bond in periplasmatic protein involved in intracellular spread, macrophage resistance and regulation two-component system like PhoPQ and pilus biogenesis | [^39–41^] |
| Regulation/stress response | DegP | *degP* | Chaperone at low temperatures and protease in high temperatures, is involved in the biogenesis of Omps and correct organization of outer membrane | [^42^] |

*a*. OmpT_P_ for plasmidial variant and OmpT_c_ for the chromosomal variant.

** Trimeric Autotransporter protein

1. Nesta B, Spraggon G, Alteri C, et al. FdeC, a Novel Broadly Conserved Escherichia coli Adhesin Eliciting Protection against Urinary Tract Infections. *MBio*. 2012;3(2):e00010-12. doi:10.1128/MBIO.00010-12

2. Lugering A, Benz I, Knochenhauer S, Ruffing M, Schmidt MA. The Pix pilus adhesin of the uropathogenic Escherichia coli strain X2194 (O2 : K- : H6) is related to Pap pili but exhibits a truncated regulatory region. *Microbiology*. 2003;149(6):1387-1397. doi:10.1099/mic.0.26266-0

3. Spurbeck RR, Stapleton AE, Johnson JR, Walk ST, Hooton TM, Mobley HLT. Fimbrial Profiles Predict Virulence of Uropathogenic Escherichia coli Strains: Contribution of Ygi and Yad Fimbriae. *Infect Immun*. 2011;79(12):4753-4763. doi:10.1128/IAI.05621-11

4. Ross BN, Rojas-Lopez M, Cieza RJ, McWilliams BD, Torres AG. The Role of Long Polar Fimbriae in Escherichia coli O104:H4 Adhesion and Colonization. Fratamico P, ed. *PLoS One*. 2015;10(10):e0141845. doi:10.1371/journal.pone.0141845

5. Korea C-G, Badouraly R, Prevost M-C, Ghigo J-M, Beloin C. Escherichia coli K-12 possesses multiple cryptic but functional chaperone-usher fimbriae with distinct surface specificities. *Environ Microbiol*. 2010;12(7):1957-1977. doi:10.1111/j.1462-2920.2010.02202.x

6. Martinez JJ, Mulvey MA, Schilling JD, Pinkner JS, Hultgren SJ. Type 1 pilus‐mediated bacterial invasion of bladder epithelial cells. *EMBO J*. 2000;19(12):2803-2812. doi:10.1093/EMBOJ/19.12.2803

7. Rendon MA, Saldana Z, Erdem AL, et al. Commensal and pathogenic Escherichia coli use a common pilus adherence factor for epithelial cell colonization. *Proc Natl Acad Sci*. 2007;104(25):10637-10642. doi:10.1073/pnas.0704104104

8. Xicohtencatl-Cortes J, Monteiro-Neto V, Saldaña Z, Ledesma MA, Puente JL, Girón JA. The type 4 pili of enterohemorrhagic Escherichia coli O157:H7 are multipurpose structures with pathogenic attributes. *J Bacteriol*. 2009;191(1):411-421. doi:10.1128/JB.01306-08

9. Totsika M, Wells TJ, Beloin C, et al. Molecular characterization of the EhaG and UpaG trimeric autotransporter proteins from pathogenic Escherichia coli. *Appl Environ Microbiol*. 2012;78(7):2179-2189. doi:10.1128/AEM.06680-11

10. Dziva F, Diemen PM van, Stevens MP, Smith AJ, Wallis TS. Identification of Escherichia coli O157 : H7 genes influencing colonization of the bovine gastrointestinal tract using signature-tagged mutagenesis. *Microbiology*. 2004;150(11):3631-3645. doi:10.1099/mic.0.27448-0

11. Samadder P, Xicohtencatl-Cortes J, Saldaña Z, et al. The *Escherichia coli ycbQRST* operon encodes fimbriae with laminin-binding and epithelial cell adherence properties in Shiga-toxigenic *E. coli* O157:H7. *Environ Microbiol*. 2009;11(7):1815-1826. doi:10.1111/j.1462-2920.2009.01906.x

12. Serra DO, Richter AM, Klauck G, Mika F, Hengge R. Microanatomy at Cellular Resolution and Spatial Order of Physiological Differentiation in a Bacterial Biofilm. *MBio*. 2013;4(2):e00103-13. doi:10.1128/MBIO.00103-13

13. Harkness RE, Olschläger T. The biology of colicin M. *FEMS Microbiol Lett*. 1991;88(1):27-42. doi:10.1111/j.1574-6968.1991.tb04955.x

14. Franzon VL, Arondel J, Sansonetti PJ. Contribution of superoxide dismutase and catalase activities to Shigella flexneri pathogenesis. *Infect Immun*. 1990;58(2):529-535. http://www.ncbi.nlm.nih.gov/pubmed/2404874. Accessed June 19, 2019.

15. Peters SE, Paterson GK, Bandularatne ESD, et al. Salmonella enterica serovar typhimurium trxA mutants are protective against virulent challenge and induce less inflammation than the live-attenuated vaccine strain SL3261. *Infect Immun*. 2010;78(1):326-336. doi:10.1128/IAI.00768-09

16. Johnson TJ, Wannemuehler YM, Nolan LK. Evolution of the iss gene in Escherichia coli. *Appl Environ Microbiol*. 2008;74(8):2360-2369. doi:10.1128/AEM.02634-07

17. Murase K, Martin P, Porcheron G, et al. HlyF Produced by Extraintestinal Pathogenic *Escherichia coli* Is a Virulence Factor That Regulates Outer Membrane Vesicle Biogenesis. *J Infect Dis*. 2016;213(5):856-865. doi:10.1093/infdis/jiv506

18. Zhuge X, Sun Y, Xue F, et al. A Novel PhoP/PhoQ Regulation Pathway Modulates the Survival of Extraintestinal Pathogenic Escherichia coli in Macrophages. *Front Immunol*. 2018;9:788. doi:10.3389/fimmu.2018.00788

19. Suzuki T, Murai T, Fukuda I, Tobe T, Yoshikawa M, Sasakawa C. Identification and characterization of a chromosomal virulence gene, vacJ, required for intercellular spreading of Shigella flexneri. *Mol Microbiol*. 1994;11(1):31-41. http://www.ncbi.nlm.nih.gov/pubmed/8145644. Accessed June 19, 2019.

20. Pilatti L, de Paiva JB, Rojas TCG, et al. The virulence factor ychO has a pleiotropic action in an Avian Pathogenic Escherichia coli (APEC) strain. *BMC Microbiol*. 2016;16(1):35. doi:10.1186/s12866-016-0654-2

21. Huang SH, Chen YH, Fu Q, et al. Identification and characterization of an Escherichia coli invasion gene locus, ibeB, required for penetration of brain microvascular endothelial cells. *Infect Immun*. 1999;67(5):2103-2109. http://www.ncbi.nlm.nih.gov/pubmed/10225861. Accessed June 19, 2019.

22. Wang Y, Kim KS. Role of OmpA and IbeB in Escherichia coli K1 Invasion of Brain Microvascular Endothelial Cells In Vitro and In Vivo. *Pediatr Res*. 2002;51(5):559-563. doi:10.1203/00006450-200205000-00003

23. Prasadarao N V, Wass CA, Stins MF, Shimada H, Kim KS. Outer membrane protein A-promoted actin condensation of brain microvascular endothelial cells is required for Escherichia coli invasion. *Infect Immun*. 1999;67(11):5775-5783. http://www.ncbi.nlm.nih.gov/pubmed/10531228. Accessed June 19, 2019.

24. Wang Y, Huang SH, Wass CA, Stins MF, Kim KS. The gene locus yijP contributes to Escherichia coli K1 invasion of brain microvascular endothelial cells. *Infect Immun*. 1999;67(9):4751-4756. http://www.ncbi.nlm.nih.gov/pubmed/10456927. Accessed June 20, 2019.

25. Horne SM, Kottom TJ, Nolan LK, Young KD. Decreased intracellular survival of an fkpA mutant of Salmonella typhimurium Copenhagen. *Infect Immun*. 1997;65(2):806-810. http://www.ncbi.nlm.nih.gov/pubmed/9009347. Accessed June 20, 2019.

26. Janakiraman A, Slauch JM. The putative iron transport system SitABCD encoded on SPI1 is required for full virulence of Salmonella typhimurium. *Mol Microbiol*. 2000;35(5):1146-1155. doi:10.1046/j.1365-2958.2000.01783.x

27. Caza M, Lépine F, Milot S, Dozois CM. Specific roles of the iroBCDEN genes in virulence of an avian pathogenic Escherichia coli O78 strain and in production of salmochelins. *Infect Immun*. 2008;76(8):3539-3549. doi:10.1128/IAI.00455-08

28. Carmel G, Coulton JW. Internal deletions in the FhuA receptor of Escherichia coli K-12 define domains of ligand interactions. *J Bacteriol*. 1991;173(14):4394-4403. doi:10.1128/jb.173.14.4394-4403.1991

29. Stumpe S, Schmid R, Stephens DL, Georgiou G, Bakker EP. Identification of OmpT as the protease that hydrolyzes the antimicrobial peptide protamine before it enters growing cells of Escherichia coli. *J Bacteriol*. 1998;180(15):4002-4006. http://www.ncbi.nlm.nih.gov/pubmed/9683502. Accessed June 20, 2019.

30. Sperandio V, Torres AG, Kaper JB. Quorum sensing *Escherichia coli* regulators B and C (QseBC): a novel two-component regulatory system involved in the regulation of flagella and motility by quorum sensing in *E. coli*. *Mol Microbiol*. 2002;43(3):809-821. doi:10.1046/j.1365-2958.2002.02803.x

31. Weigel WA, Demuth DR. QseBC, a two-component bacterial adrenergic receptor and global regulator of virulence in *Enterobacteriaceae* and *Pasteurellaceae*. *Mol Oral Microbiol*. 2016;31(5):379-397. doi:10.1111/omi.12138

32. Nishino K, Yamaguchi A. Analysis of a Complete Library of Putative Drug Transporter Genes in Escherichia coli. *J Bacteriol*. 2001;183(20):5803-5812. doi:10.1128/JB.183.20.5803-5812.2001

33. Daniels JJ, Autenrieth IB, Ludwig A, Goebel W. The gene slyA of Salmonella typhimurium is required for destruction of M cells and intracellular survival but not for invasion or colonization of the murine small intestine. *Infect Immun*. 1996;64(12):5075-5084. http://www.ncbi.nlm.nih.gov/pubmed/8945549. Accessed June 20, 2019.

34. Buchmeier N, Bossie S, Chen CY, Fang FC, Guiney DG, Libby SJ. SlyA, a transcriptional regulator of Salmonella typhimurium, is required for resistance to oxidative stress and is expressed in the intracellular environment of macrophages. *Infect Immun*. 1997;65(9):3725-3730. http://www.ncbi.nlm.nih.gov/pubmed/9284144. Accessed June 20, 2019.

35. Linehan SA, Rytkönen A, Yu X-J, Liu M, Holden DW. SlyA regulates function of Salmonella pathogenicity island 2 (SPI-2) and expression of SPI-2-associated genes. *Infect Immun*. 2005;73(7):4354-4362. doi:10.1128/IAI.73.7.4354-4362.2005

36. Chan K, Kim CC, Falkow S. Microarray-based detection of Salmonella enterica serovar Typhimurium transposon mutants that cannot survive in macrophages and mice. *Infect Immun*. 2005;73(9):5438-5449. doi:10.1128/IAI.73.9.5438-5449.2005

37. Korch SB, Henderson TA, Hill TM. Characterization of the hipA7 allele of Escherichia coli and evidence that high persistence is governed by (p)ppGpp synthesis. *Mol Microbiol*. 2003;50(4):1199-1213. doi:10.1046/j.1365-2958.2003.03779.x

38. Boehm A, Steiner S, Zaehringer F, et al. Second messenger signalling governs *Escherichia coli* biofilm induction upon ribosomal stress. *Mol Microbiol*. 2009;72(6):1500-1516. doi:10.1111/j.1365-2958.2009.06739.x

39. Totsika M, Heras B, Wurpel DJ, Schembri MA. Characterization of two homologous disulfide bond systems involved in virulence factor biogenesis in uropathogenic Escherichia coli CFT073. *J Bacteriol*. 2009;191(12):3901-3908. doi:10.1128/JB.00143-09

40. Yu J, Oragui EE, Stephens A, Kroll JS, Venkatesan MM. Inactivation of DsbA alters the behaviour of *Shigella flexneri* towards murine and human-derived macrophage-like cells. *FEMS Microbiol Lett*. 2001;204(1):81-88. doi:10.1111/j.1574-6968.2001.tb10867.x

41. Lippa AM, Goulian M. Perturbation of the oxidizing environment of the periplasm stimulates the PhoQ/PhoP system in Escherichia coli. *J Bacteriol*. 2012;194(6):1457-1463. doi:10.1128/JB.06055-11

42. Purdy GE, Hong M, Payne SM. Shigella flexneri DegP facilitates IcsA surface expression and is required for efficient intercellular spread. *Infect Immun*. 2002;70(11):6355-6364. doi:10.1128/iai.70.11.6355-6364.2002
