# Supplementary figures and images for: Virulence Potential of a Multidrug-Resistant Escherichia coli Strain Belonging to the Emerging Clonal Group ST101-B1 Isolated from Bloodstream Infection
Source: Microorganisms. 2020 May 30;8(6):827. doi: 10.3390/microorganisms8060827 (PMC7355805; doi:10.3390/microorganisms8060827)

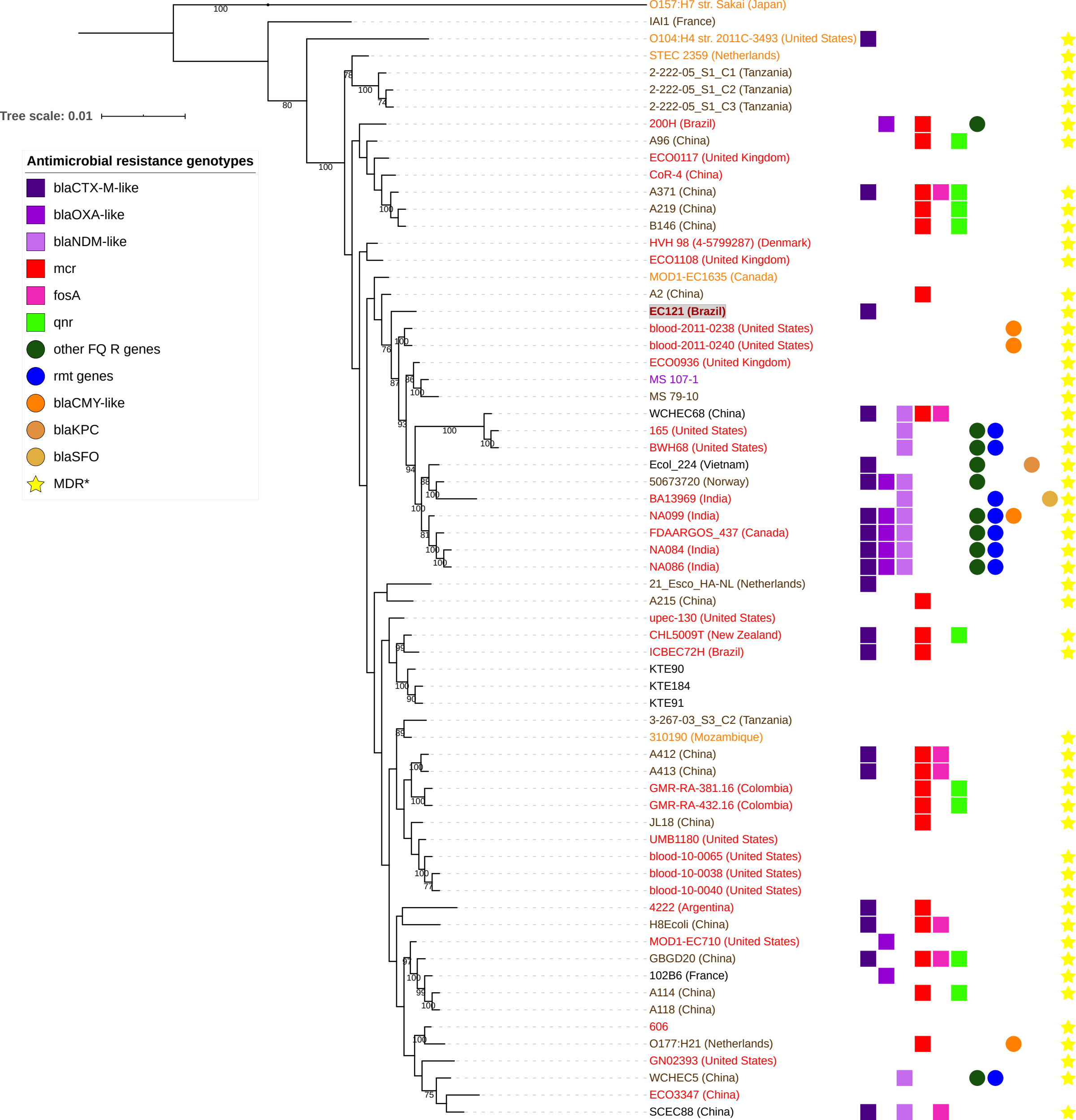

Supplement: Supplementary file 1 [file microorganisms-08-00827-s001.zip › Suppl/Figure S1 Phylogenetic tree S1.pdf]

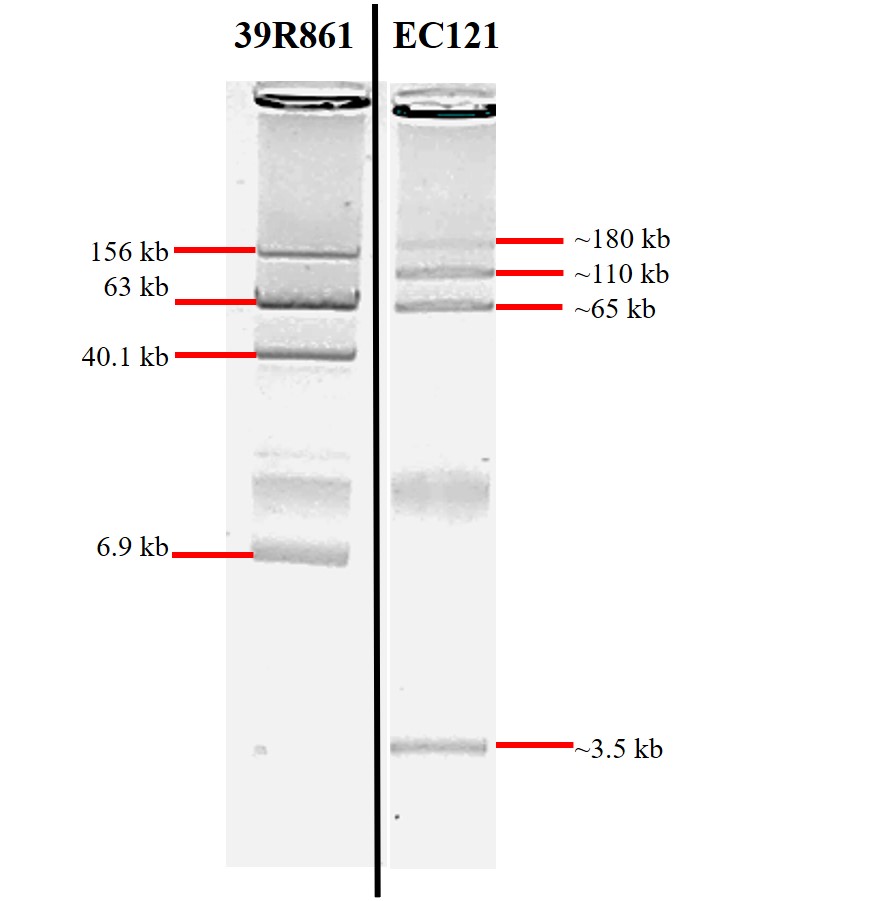

Supplement: Supplementary file 1 [file microorganisms-08-00827-s001.zip › Suppl/Figure S2 Plasmid.jpg]

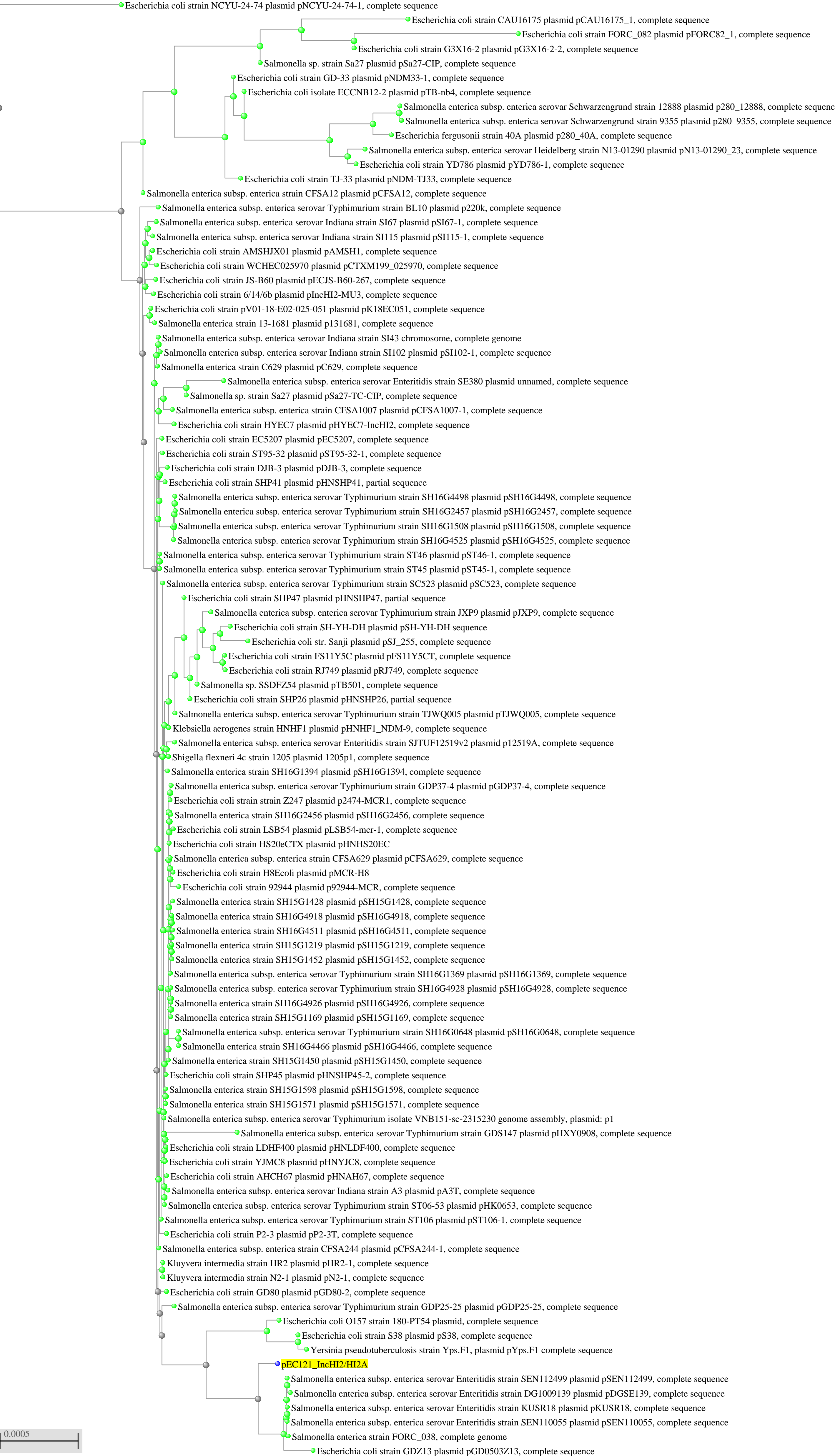

Supplement: Supplementary file 1 [file microorganisms-08-00827-s001.zip › Suppl/Figure S3 IncHI2 plasmid phylogenetic tree.pdf]

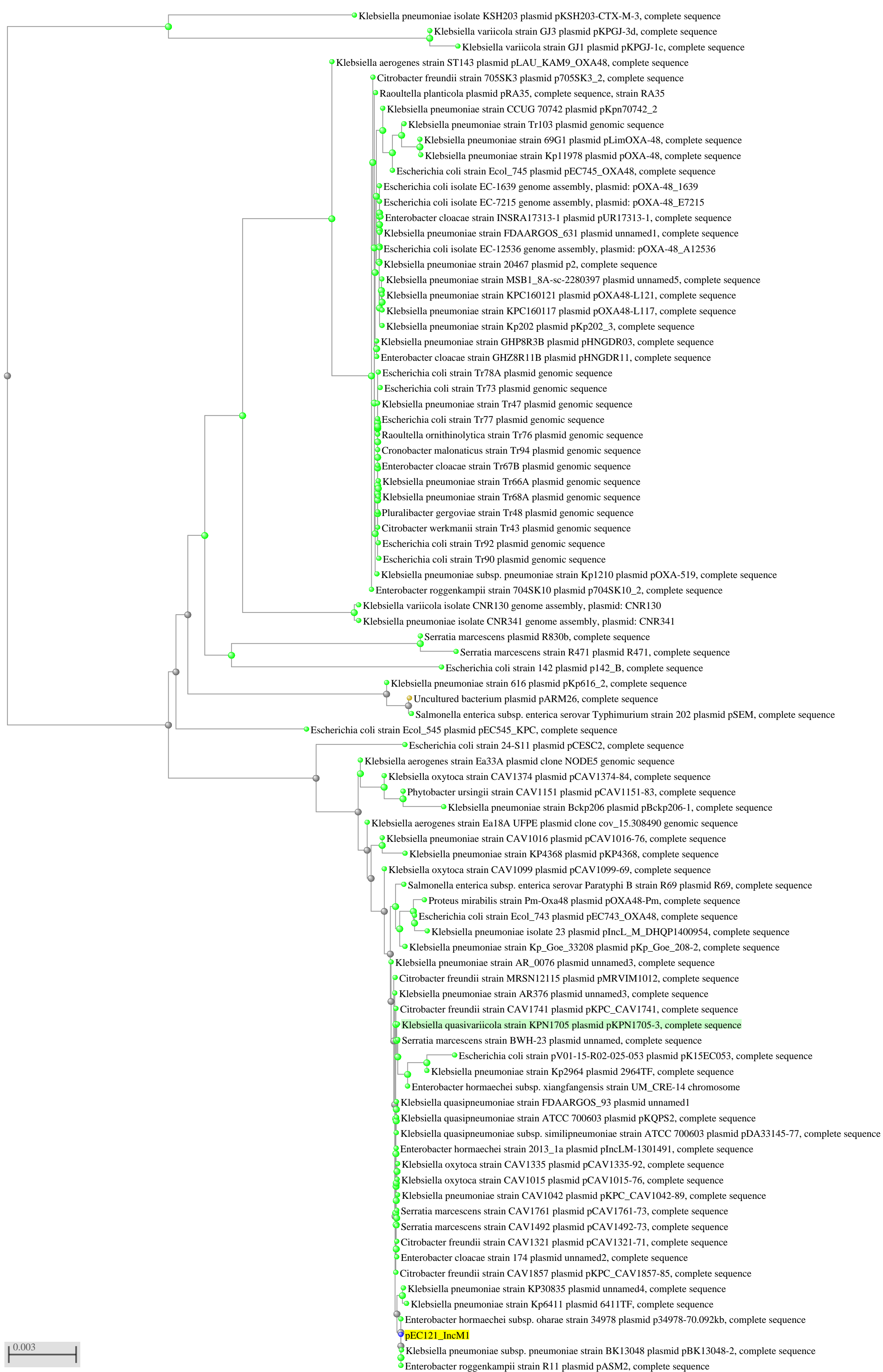

Supplement: Supplementary file 1 [file microorganisms-08-00827-s001.zip › Suppl/Figure S4 IncM1 plasmid phylogenetic tree.pdf]
